# Photobiomodulation and different macrophages phenotypes during muscle tissue repair

**DOI:** 10.1111/jcmm.13757

**Published:** 2018-07-19

**Authors:** Nadhia H. C. Souza, Raquel A. Mesquita‐Ferrari, Maria Fernanda S. D. Rodrigues, Daniela F. T. da Silva, Beatriz G. Ribeiro, Agnelo N. Alves, Mónica P. Garcia, Fábio D. Nunes, Evaldo M. da Silva Junior, Cristiane M. França, Sandra K. Bussadori, Kristianne P. S. Fernandes

**Affiliations:** ^1^ Postgraduate Program in Rehabilition Sciences Nove de Julho University (UNINOVE) São Paulo Brazil; ^2^ Postgraduate Program in Biophotonics Applied to Health Sciences Nove de Julho University (UNINOVE) São Paulo Brazil; ^3^ Departament of Oral Pathology School of Dentistry University of São Paulo (FOUSP) São Paulo Brazil

**Keywords:** macrophages, muscle, photobiomodulation, regeneration, regenerative medicine

## Abstract

Macrophages play a very important role in the conduction of several regenerative processes mainly due to their plasticity and multiple functions. In the muscle repair process, while M1 macrophages regulate the inflammatory and proliferative phases, M2 (anti‐inflammatory) macrophages direct the differentiation and remodelling phases, leading to tissue regeneration. The aim of this study was to evaluate the effect of red and near infrared (NIR) photobiomodulation (PBM) on macrophage phenotypes and correlate these findings with the repair process following acute muscle injury. Wistar rats were divided into 4 groups: control; muscle injury; muscle injury + red PBM; and muscle injury + NIR PBM. After 2, 4 and 7 days, the tibialis anterior muscle was processed for analysis. Macrophages phenotypic profile was evaluated by immunohistochemistry and correlated with the different stages of the skeletal muscle repair by the qualitative and quantitative morphological analysis as well as by the evaluation of *IL‐6*,*TNF‐*α and *TGF‐*β mRNA expression. Photobiomodulation at both wavelengths was able to decrease the number of CD68^+^ (M1) macrophages 2 days after muscle injury and increase the number of CD163^+^ (M2) macrophages 7 days after injury. However, only NIR treatment was able to increase the number of CD206^+^ M2 macrophages (Day 2) and TGF *‐*β mRNA expression (Day 2, 4 and 7), favouring the repair process more expressivelly. Treatment with PBM was able to modulate the inflammation phase, optimize the transition from the inflammatory to the regeneration phase (mainly with NIR light) and improve the final step of regeneration, enhancing tissue repair.

## 1 INTRODUCTION

Acute muscle injuries provide a good model for the study of the modulating effect of immune cells on the tissue repair process.^1^ Immediately after an acute injury, muscle tissue undergoes the rapid invasion of inflammatory cells, mainly neutrophils and macrophages.^1^ Macrophages constitute the majority of intramuscular leucocytes and, besides removing tissue debris through phagocytosis, these cells synthesize growth factors, chemokines and cytokines that modulate all phases of muscle repair.^1^, ^2^

Under the microenvironment stimuli provided mainly by infiltrated neutrophils (ie, presence of Th1 mediators, such as *IFN‐*γ and *TNF‐*α) in the initial steps after an acute muscle injury, macrophages are activated and acquire a proinflammatory phenotype, classically known as M1 (CD68^high^, CD206^−^ and CD163^−^) and characterized by enhanced phagocytic activity and production of proinflammatory mediators as IL‐1β, IL‐6, TNF‐α, IL‐12 and IL‐23.^1^, ^3^, ^4^, ^5^ Other mediators released by M1 macrophages, such as IL‐6, IL‐1, VEGF and IL‐13, also stimulate angiogenesis and the proliferation of myogenic precursor cells.^1^, ^4^, ^5^ M1 cell surface marker CD68 is a receptor for oxidized low‐density lipoproteins that activate phagocytosis and increase the production of proinflammatory cytokines when specifically connected. Normal muscle tissue does not express CD68^+^ macrophages.^3^, ^4^

After approximately 3 days, other macrophage phenotypes, identified as M2 (CD68^low^, CD206^+^ and CD163^+^) or alternatively activated macrophages, become more numerous in the damaged tissue and persist until 7 days after injury.^1^ M2 macrophages produce anti‐inflammatory cytokines and growth factors as *TGF‐*β and *IL‐10* as well as enzymes that are important to angiogenesis, fibroblast proliferation and the differentiation of myogenic precursor cells.^1^, ^4^, ^5^ M2 macrophage surface marker CD206 is a mannose receptor that internalizes sugar moieties on molecules in inflamed tissue, such as myeloperoxidase.^3^ CD163 is a specific receptor for hemoglobin and haptaglobin complexes.^3^ Specific binding to the both receptors triggers the expression of anti‐inflammatory cytokines, such as *IL‐10* and *TGF‐*β, leading to the deactivation of M1 macrophages^1^, ^3^, ^4^ and enabling the predominance of M2 macrophages at the injury site during the transition from the proliferative stage to the differentiation and growth stage of myogenesis.^1^, ^3^, ^4^

It is well accepted that although there are different degrees of differentiation between the populations of macrophages that inhabit the muscle tissue after an injury, the coordinated activation of proinflammatory or anti‐inflammatory macrophage predominance in each step of the muscle repair process is essential to the resolution of the inflammatory process and regeneration of the muscle tissue.^1^, ^4^, ^5^

The modulation of macrophage plasticity is considered so important that macrophage‐based therapeutic interventions are currently emphasized in regenerative medicine to improve the healing process and avoid undesirable effects associated with altered macrophage function.^6^, ^7^ Among the therapeutic interventions for the treatment of muscle injuries, photobiomodulation (PBM) has been extensively investigated (for review, see^8^). Photobiomodulation consists of the use of low‐power non‐thermal light using a source (such as laser or LED) to modulate inflammation and healing (see^9^, ^10^, ^11^ and references therein). The most common spectral regions used in PBM are the red (600‐700 nm) and near infrared (NIR, 780‐110 nm) wavelengths,^9^, ^10^, ^11^ both of which achieve greater tissue penetration compared to other wavelengths due to the lower absorption and scattering by tissue chromophores.^9^, ^10^, ^11^

Regarding the muscle tissue repair process, the use of NIR PBM is more common, but both red and NIR therapies are reported to decrease myonecrosis and the infiltration of inflammatory cells^12^, ^13^ as well as increase the number of immature muscle fibres, leading to better organized muscle tissue.^8^, ^12^, ^13^, ^14^, ^15^ In a time‐dependent manner, red and NIR therapies are also able to modulate the gene expression of mediators, such as *TNF‐*α*,* 15, 16 *IL1‐*β*,* 17 *IL‐6* 18 and *TGF‐*β^16^, ^19^ as well as genes involved in the differentiation of myogenic stem cells, such as *MyoD* 18, 20 and *myogenin* 18, 20 during the muscle repair process.

As macrophages are the main source of cytokines, chemokines and growth factors that guide muscle repair, it is important to investigate whether PBM modulates the different macrophage phenotypes during the progression of the repair process. Thus, the aim of the present study was to compare the effect of red and NIR PBM on the muscle repair process following an acute injury and correlate the findings with the presence of macrophage phenotypes, mRNA expression of *IL‐6*,* TNF‐*α and *TGF‐*β, and the evolution of tissue repair after different experimental periods.

## 2 MATERIALS AND METHODS

All animal procedures were performed in accordance with the guidelines of the National Council for the Control of Animal Experimentation and received approval from the Animal Research Ethics Committee (certificate number: 0017/2014). Fifty male Wistar rats (*Rattus norvegicus*: var. *albinus*, Rodentia, Mammalia) were kept under controlled temperature (22°C) and relative humidity (40%), with a 12‐hour light/dark cycle. The animals were offered solid ration and water ad libitum throughout the experimental period. The 2‐month old (200 ± 15 g) animals were randomly divided into 4 experimental groups: (i) control group (n = 5, not subjected to injury or PBM); (ii) injury group (n = 15, subjected to cryoinjury and not treated with PBM); (iii) injury + PBM 660 nm group (n = 15, cryoinjury and treatment with red PBM λ = 660 nm); and (iv) injury + PBM 780 nm (n = 15, cryoinjury and treatment with NIR PBM λ = 780 nm). Animals in Groups 2, 3 and 4 (n = 5) and Group 1 (n = 1) were euthanized on Days 2, 4 and 7 following the induction of injury for analysis.

### 2.1 Injury procedure

The cryoinjury procedure was performed using a previously described method.^16^, ^18^ The surgical procedures were performed under anesthesia with 10% ketamine HCl (Dopalen; Vetbrands, São Paulo, Brazil) and 2% xylazine (Anasedan; Vetbrands) (100 and 10 mg/kg, respectively). The tibialis anterior (TA) muscle was surgically exposed by a 15‐mm‐long longitudinal skin incision over the central portion of the muscle. The cryoinjury procedure consisted of applying the flat end of a metal rod (3 mm in diameter), which had previously been cooled in liquid nitrogen, directly to the ventral surface of the exposed muscle for 10 seconds. After the area had thawed, the procedure was repeated for an additional 10 seconds, followed by the suturing of the incision (Figure S2A).

### 2.2 PBM treatment protocol

Photobiomodulation treatment was initiated 2 hours after the cryoinjury procedure and was performed daily with a 24‐hour interval between sessions.^16^, ^17^, ^18^ Photobiomodulation was performed with aluminium‐gallium‐indium‐phosphide (AlGaInP) and aluminium‐gallium‐arsenide (AlGaAs) diode lasers (Twin Laser; MM Optics, São Carlos, SP, Brazil) operating at a wavelength of 660 and 780 nm, respectively. The dosimetric parameters are described in Table 1. The laser beam was applied in contact with the skin surface over the cryoinjury area at an angle of 90° between the emitter and skin to prevent reflection. Irradiation was applied to 8 points (Figure S2B) surrounding the sutured area.^12^, ^16^, ^17^, ^18^ After 2, 4 and 7 days of treatment, the animals were weighed and euthanized with an overdose of anesthesia (240 mg/kg of ketamine and 30 mg/kg of xylazine). The TA muscle was removed for morphological and immunohistochemical analysis as well as for mRNA expression evaluation.

**Table 1:** PBM treatment parameters

Active medium	AlGaInP	AlGaAs
Wavelength	660	780
Beam area	0.04 cm^2^	0.04 cm^2^
Power output	70 mW	70 mW
Power density	1750 mW/cm^2^	1750 mW/cm^2^
Energy density	25.025 J/cm^2^	25.025 J/cm^2^
Energy per point	1 J	1 J
Total points	8	8
Time per point	15 s	15 s
Total time	120 s	120 s
Total energy	8 J	8 J

PBM, photobiomodulation.

### 2.3 Morphological analysis

Muscle samples were fixed in 10% buffered formalin (pH 7.4), embedded in paraffin and sectioned with a microtome (Leica RM2125, Nussloch, Germany). The sections were stained with hematoxylin‐eosin (H&E) and examined under a light microscope (Zeiss, Axioplan 2, Germany). Five areas representing at least 70% of the injury were photographed with a 20× objective (magnification: 200×) in each section. Morphological aspects relevant to muscle repair, such as myonecrosis, inflammatory infiltrate, blood vessels and immature muscle fibres, were quantitatively and qualitatively evaluated using the Image J cell count software plug‐in (National Institutes of Health, USA) by an experienced pathologist with no knowledge of the experimental groups.^13^, ^21^, ^22^ The results of the 5 areas of each section were summed. Three samples from each group were examined and the data were subjected to statistical analysis.

### 2.4 Immunohistochemical analysis of macrophage phenotypes

Muscle samples were fixed in 10% buffered formalin (pH 7.4), embedded in paraffin, cut in 3‐μm sections and placed on slides with a 2% solution of 3‐aminopropyltriethylsilane (Sigma‐Aldrich, St. Louis, MO, USA). De‐paraffinization was performed with xylene and the samples were immersed in a graded series of ethanol/water concentrations (100%, 90%, 70% and 50%). After rinsing with Tris‐buffered saline (TBS, pH 7.4), endogenous peroxide activity was blocked with 3% hydrogen peroxide for 45 minutes. For the analysis of CD68 and CD206 expression, antigen retrieval was performed by incubating the slides with 100 mmol/L citrate buffer (pH 6.0) for 30 minutes at 95°C. For the evaluation of CD163 expression, antigen retrieval was performed with a 0.25% trypsin solution for 20 minutes at 37°C. After washing, non‐specific binding sites were blocked using 3% goat serum for 20 minutes at room temperature. Slides were incubated with the primary antibodies anti‐CD68 (1:1500; Abcam Inc, Cambridge, MA, USA), anti‐CD206 (1:2500; Abcam Inc) and anti‐CD163 (1:50; BIO‐RAD, Hercules, CA, USA), diluted in a diluent solution (Spring Bioscience, Pleasanton, CA, USA) and incubated at 4°C overnight. After incubation, tissue sections were washed in TBS and incubated with the secondary antibody Histofine^®^ Simple Stain MAX PO (Nichirei Biosciences Inc., Tsukiji, Chuo‐Ku, Tokyo, Japan) for 30 minutes at 37°C. The reactions were revealed by incubating the sections with the 3,3‐diaminobenzidine chromogen (DAB; Sigma‐Aldrich Chemical, Steinheim, Germany) and counterstained with Mayer's hematoxylin. Negative controls were obtained by substituting the primary antibody with non‐immune serum. Control slides from animals not subjected to injury or treatment were subjected to the same procedures. Five samples were analyzed for each animal and a minimum of 10 images were captured using a light microscope (Zeiss, Axioplan 2) with a 40× objective (magnification: 400×). The images were evaluated by an experienced pathologist with no knowledge of the experimental groups. The number of cells positive for CD68, CD206 and CD163 was manually counted using the Image J cell count software plug‐in (National Institutes of Health) and the data were subjected to statistical analysis.

### 2.5 cDNA synthesis and real‐time PCR analysis

Total RNA was isolated from TA muscles using cold Trizol reagent (Invitrogen, CA, USA), following the manufacturer's instructions. RNA quantity and integrity were assessed using spectrophotometry (NanoDrop 2000; Thermo Scientific, USA) and electrophoresis using 1% agarose gel stained with ethidium bromide, respectively. One microgram of total RNA was incubated with DNAse I (Invitrogen, Brazil) and reversed transcribed to single‐stranded cDNA using the High Capacity cDNA Reverse Transcription Kit (Applied Biosystems, Foster City, CA, USA), following the manufacturer's instructions. The reactions conditions were 20°C for 10 minutes, 42°C for 45 minutes and 95°C for 5 minutes. Real‐time PCR was performed in a ABI7500 Fast Real‐Time System (ABI Prism; Applied Biosystems) with Power SYBR Green I Dye. All qRT‐PCR reactions were performed in a total volume of 10 μL, containing 1 μL of cDNA sample, 10 pmol of each primer (400 nmol/L) and 5 μL of SYBR Green Master Mix^®^ (Applied Biosystems). Thermal cycling was conducted starting with 50°C for 2 minutes and 95°C for 10 minutes, followed by 40 amplification cycles of 95°C for 10 seconds and 60°C for 1 minute. Specific primers for rat *TNF‐*α (forward 5′‐AAATGGGCTCCCTCTATCAGTTC‐3′; reverse 5′‐TCTGCTTGGTGGTTTGCTACGAC‐3′; GenBank accession # X66539), *TGF‐*β (forward 5′‐CCCCTGGAAAGGGCTCAACAC‐3′; reverse 5′‐TCCAACCCAGGTCCTTCCTAAAGTC‐3′; GenBank accession # NM021578.2) and *IL‐6* (forward 5′‐TCCAGTTGCCTTCTTGGGAC‐3′; reverse 5′‐GTGTAATTAAGCCTCCGACTTG‐3′: GenBank accession # NM 031168.1) were used. Glyceraldehyde 3‐phosphate dehydrogenase (GAPDH; 5′‐TGCACCACCAACTGCTTAGC‐3′; reverse GCCCCACGGCCATCA; GenBank accession # NM 017008) was used as the endogenous control. Quantification was performed using the 2^−∆∆CT^ method^23^ and the control group was used as reference.

### 2.6 Statistical analysis

Statistical analysis was performed with GraphPad Prism 7 software (San Diego, CA, USA). Data were expressed as mean values ± standard error of the mean (SEM). Statistical differences were evaluated using 1‐way analysis of variance (ANOVA) followed by Tukey's post hoc test. Results were considered significant when *P* < .05.

## 3 RESULTS

### 3.1 Morphological analysis of muscle tissue repair

**Figure 1:**
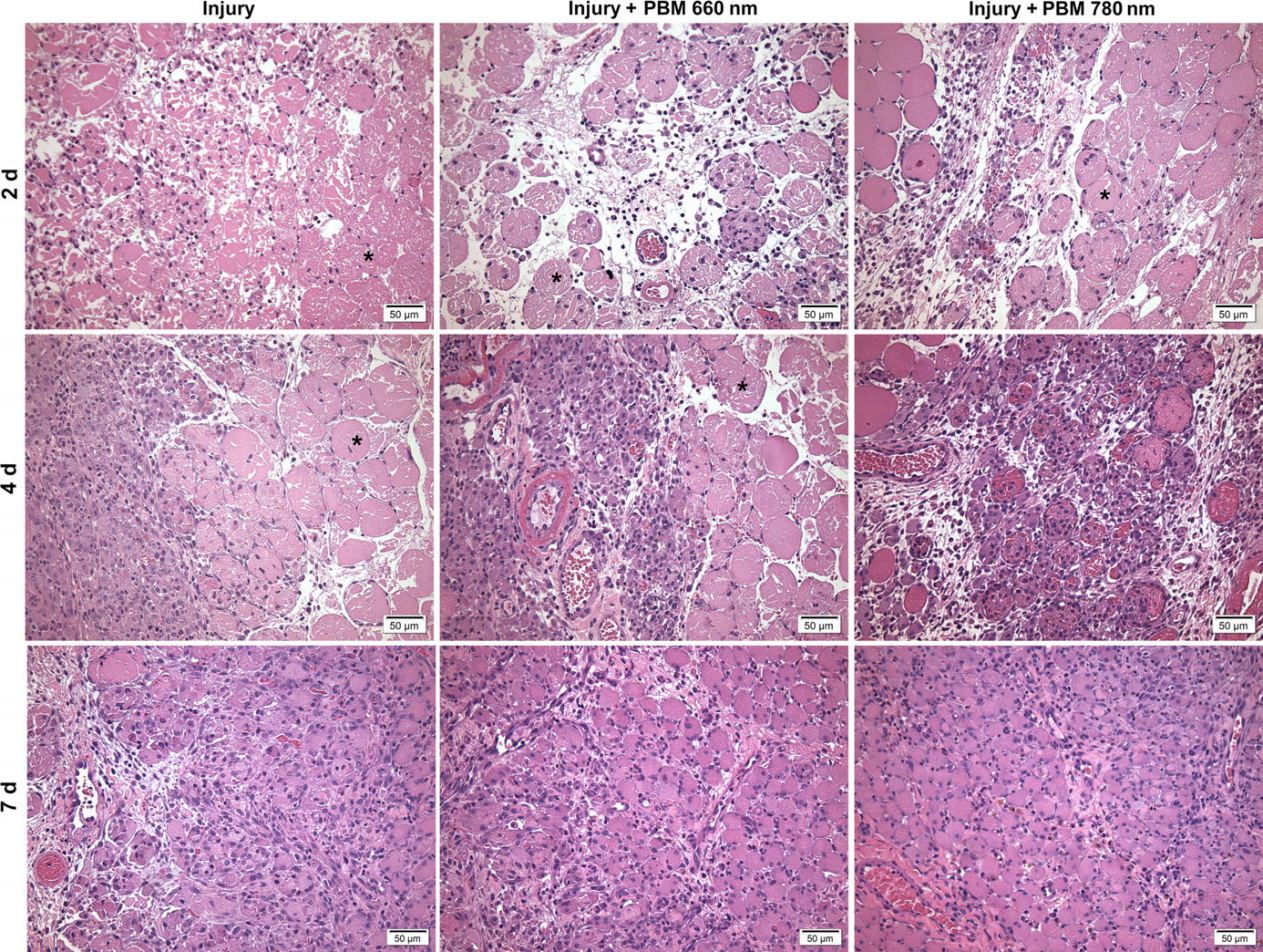
Morphological evaluation of injury, injury + PBM 660 nm and injury + PBM 780 nm groups after 2, 4 and 7 d. The injury group exhibited more myonecrosis (*) and inflammatory cells in comparison to the PBM groups. PBM 780 nm was associated with largest number of mature vessels. Both PBM treatments were able to promote the formation of immature muscle fibres (H&E staining, original magnification: 200×). PBM, photobiomodulation

**Figure 2:**
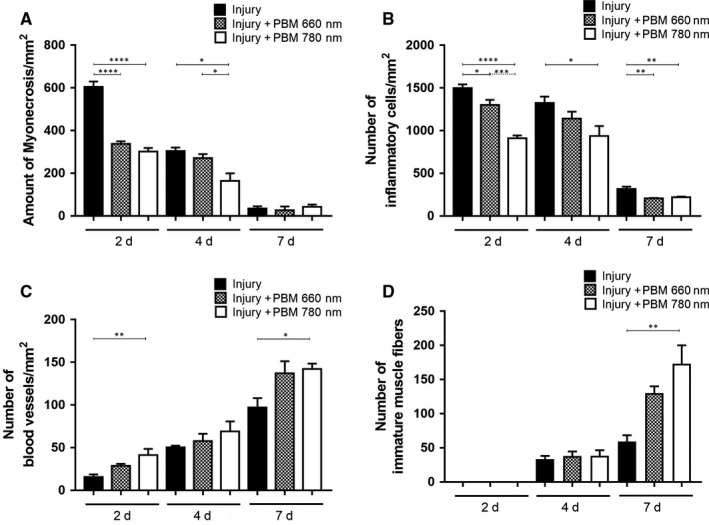
Quantitative analysis of morphological parameters evaluated in injury, PBM 660 nm and PBM 780 nm groups after 2, 4 and 7 d. Myonecrosis (A), number of inflammatory cells (B), number of blood vessels (C) and number of immature muscle fibres (D). Data expressed as mean ± SEM. (ANOVA/Tukey's test; *P < .05; **P < .01; ***P < .001; ****P < .0001 compared to the injury group without PBM). PBM, photobiomodulation

Figure 1 shows the morphological aspects of each group in each period evaluated. The control group had skeletal muscle with normal morphology (polygonal fibres with multiple peripheral nuclei) and no signs of injury (Figure S1). Figure 2 displays the results of the quantitative analysis of the parameters described above.

#### 3.1.1 Myonecrosis

All groups subjected to injury exhibited a higher level of myonecrosis on Day 2, which decreased over time. The injury group exhibited significant extensive areas of myonecrosis in comparison to the PBM 660 nm and PBM + 780 nm groups after 2 days (*P* < .0001; Figure 1). Myonecrosis was also accompanied by focal areas of oedema in the injury group. No significant differences were found between the groups treated with PBM. On Day 4, the group subjected to PBM 780 nm demonstrated a significant decrease in myonecrosis compared to both the injury group and PBM 660 nm group (*P* = .0109 and *P* = .0373, respectively; Figure 2A). Seven days after treatment, a low degree of myonecrosis was found in all groups, with no significant differences among the groups (Figure 2A).

#### 3.1.2 Inflammatory cells

No inflammatory cells were found throughout the experimental period in the control group (Figure S1). On Day 2 (Figure 1), an acute inflammatory process was found in all groups subjected to cryoinjury, but the PBM 660 nm and PBM 780 nm groups had a significant decrease in the number of inflammatory cells in comparison to the injury group (*P* = .0460 and *P* < .0001, respectively). Moreover, the reduction was significantly more pronounced in the PBM 780 nm compared to the PBM 660 nm group (*P* = .0008). On Day 4 (Figure 2B), no significant difference was found between the 2 PBM groups, but only the PBM 780 nm group had a significant lower number of inflammatory cells compared to the injury group (*P* = .0452). On Day 7 (Figure 2B), both PBM treated groups had a significantly lower number of inflammatory cells compared to the injury group (*P* = .0023 and *P* = .0054) and no significant difference was found between the PBM groups.

#### 3.1.3 Blood vessels

Well‐vascularized muscle tissue was found in the control group (Figure S1). An increased number of blood vessels was found during the phases of muscle repair and was evident on Day 7 in all groups subjected to cryoinjury, indicating the repair process (Figure 2C). On Days 2 and 7, only the PBM 780 nm group exhibited increased number of blood vessels in relation to the injury group, indicating the occurrence of a more preserved tissue (*P* = .0077 and *P* = .0277, respectively; Figure 2C). No differences were found between the injury group and PBM 660 nm group or between the 2 PBM groups.

#### 3.1.4 Immature muscle fibres

As expected, no immature fibres were found on Day 2 in any of the groups subjected to cryoinjury, as the initial phase of tissue repair was characterized by an acute inflammatory process (Figure 2D). On Days 4 and 7, the emergence of numerous immature muscle fibres with central nuclei occurred, evidencing tissue regeneration. No difference was found among the groups on Day 4. On Day 7, however, the PBM 660 nm and PBM 780 nm groups exhibited an increase in the number of immature muscle fibres in comparison to the injury group, which was more evident in the PBM 780 nm group (*P* = .0089; Figure 2D).

Taken together, these results demonstrate that PBM, specifically at 780 nm, favours muscle tissue regeneration by decreasing the acute inflammation process in the initial steps of tissue repair, minimizing myonecrosis, improving the blood supply and enhancing the proliferation of muscle cells, which are essential to the success of the muscle repair process.

### 3.2 Characterization of macrophage phenotypes during muscle tissue repair

#### 3.2.1 CD68^+^ macrophages

**Figure 3:**
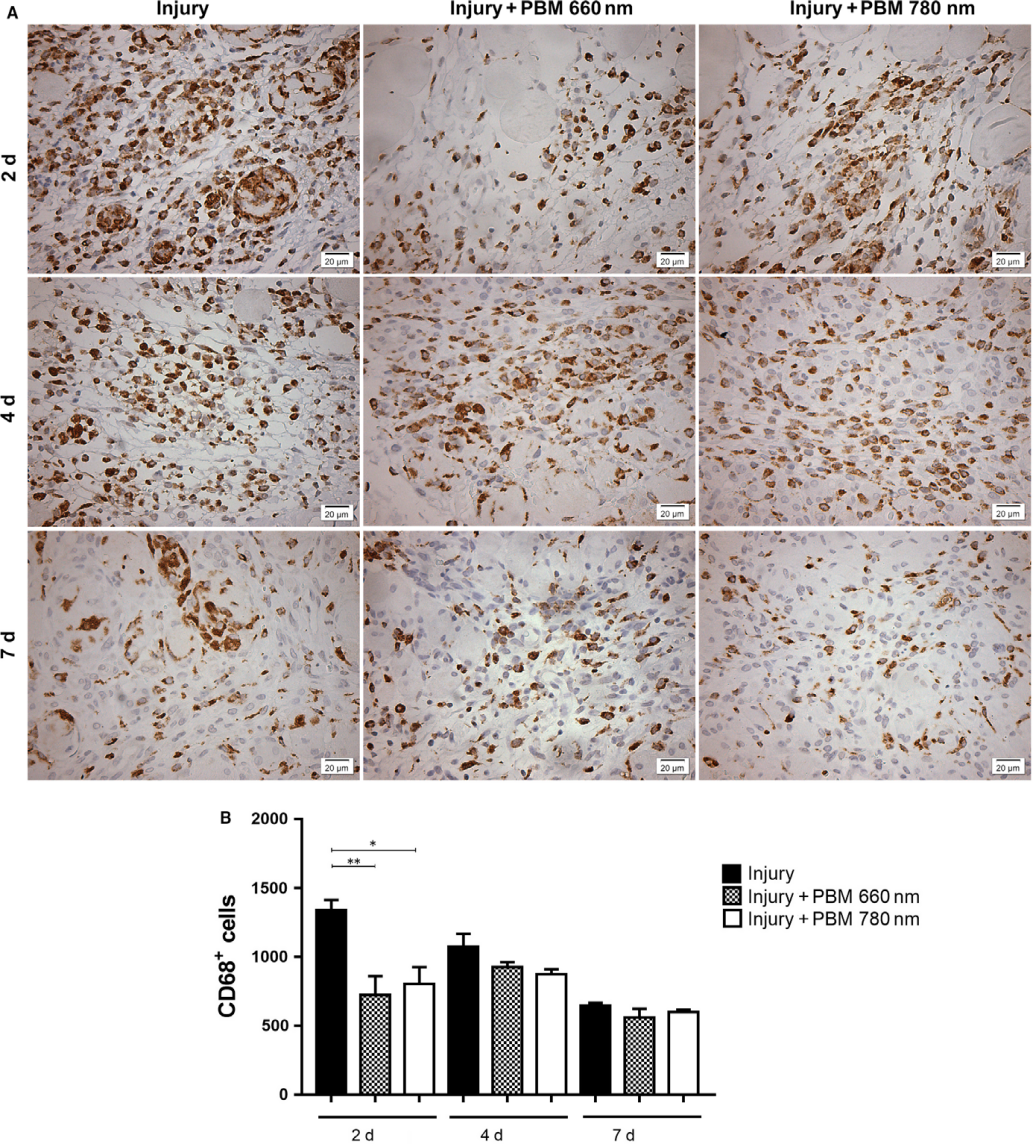
Immunohistochemical evaluation of CD68^+^ macrophages infiltration. Representative images (A) and quantitative analysis (B). The number of CD68^+^ cells was evaluated in injured muscle with or without PBM treatment in 3 different periods (2, 4 and 7 d). A significant decrease was found in the PBM 660 and 780 nm groups in comparison to the injury group (*P* < .01 and *P* < .05, respectively) on Day 2 (Original magnification: 400×). Data expressed as mean ± SEM. ANOVA/Tukey's test; *P < .05; **P < .01. PBM, photobiomodulation

No CD68^+^ macrophages were observed throughout the experimental period in the muscle tissue in the control group (Figure S1B). In the injury group, the number of CD68^+^ macrophages decreased between Days 2 and 7. On Day 2, both PBM groups exhibited a lower number of CD68^+^ macrophages in comparison to the injury group (*P* = .0073 and *P* = .0176, respectively; Figure 3). On Days 4 and 7 after injury, the number of CD68^+^ macrophages was similar among all groups (with or without PBM). No significant differences were found between the PBM 660 and 780 nm groups in any of the evaluation periods (Figure 3).

#### 3.2.2 CD206^+^ macrophages

**Figure 4:**
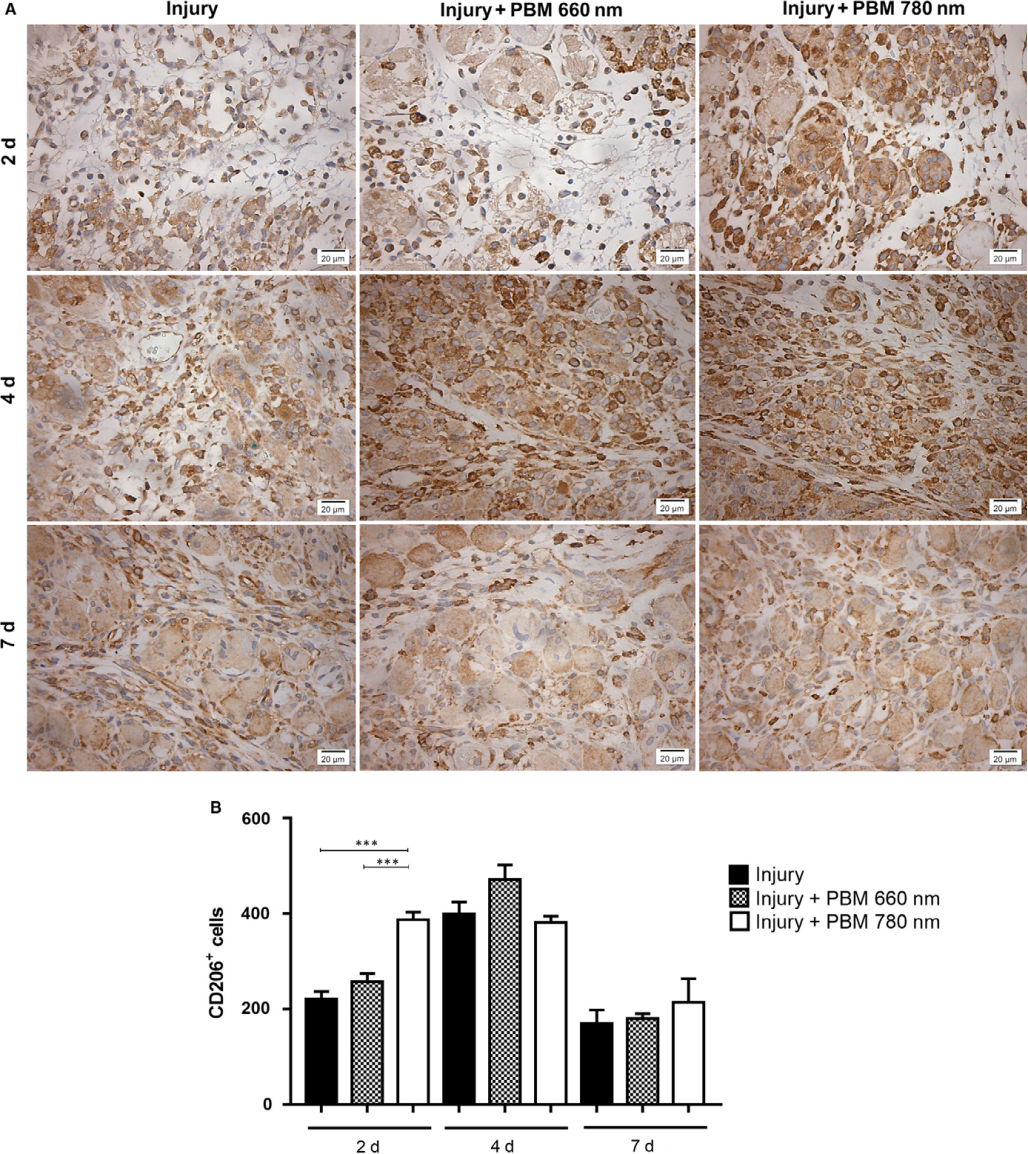
Immunohistochemical evaluation of CD206^+^ macrophages. Representative images (A) and quantitative analysis (B). The number of CD206^+^ cells was evaluated in injured muscle with or without PBM treatment in different periods (2, 4 and 7 d). A significant increase was found in the PBM 780 nm group in comparison to both the injury and PBM 660 nm groups (*P* < .001 and .001, respectively) (Original magnification: 400×). Data expressed as mean ± SEM. ANOVA/Tukey's test; ***P < .001. PBM, photobiomodulation

No CD206^+^ macrophages were observed in the control group (Figure S1B). In the injury group, the number of CD206^+^ macrophages reached a peak on Day 4 (Figure 4). The PBM 780 nm group showed an increase in the infiltration of CD206^+^ macrophages on Day 2 compared to the injury group and PBM 660 nm group (*P* = .0001 and *P* = .0009, respectively; Figure 4). The number of CD206^+^ macrophages remained high until Day 4 in the PBM 780 nm. Four and 7 days after injury, no significant differences were found in the number of CD206^+^ macrophages among the groups subjected to cryoinjury (Figure 4).

#### 3.2.3 CD163^+^ macrophages

**Figure 5:**
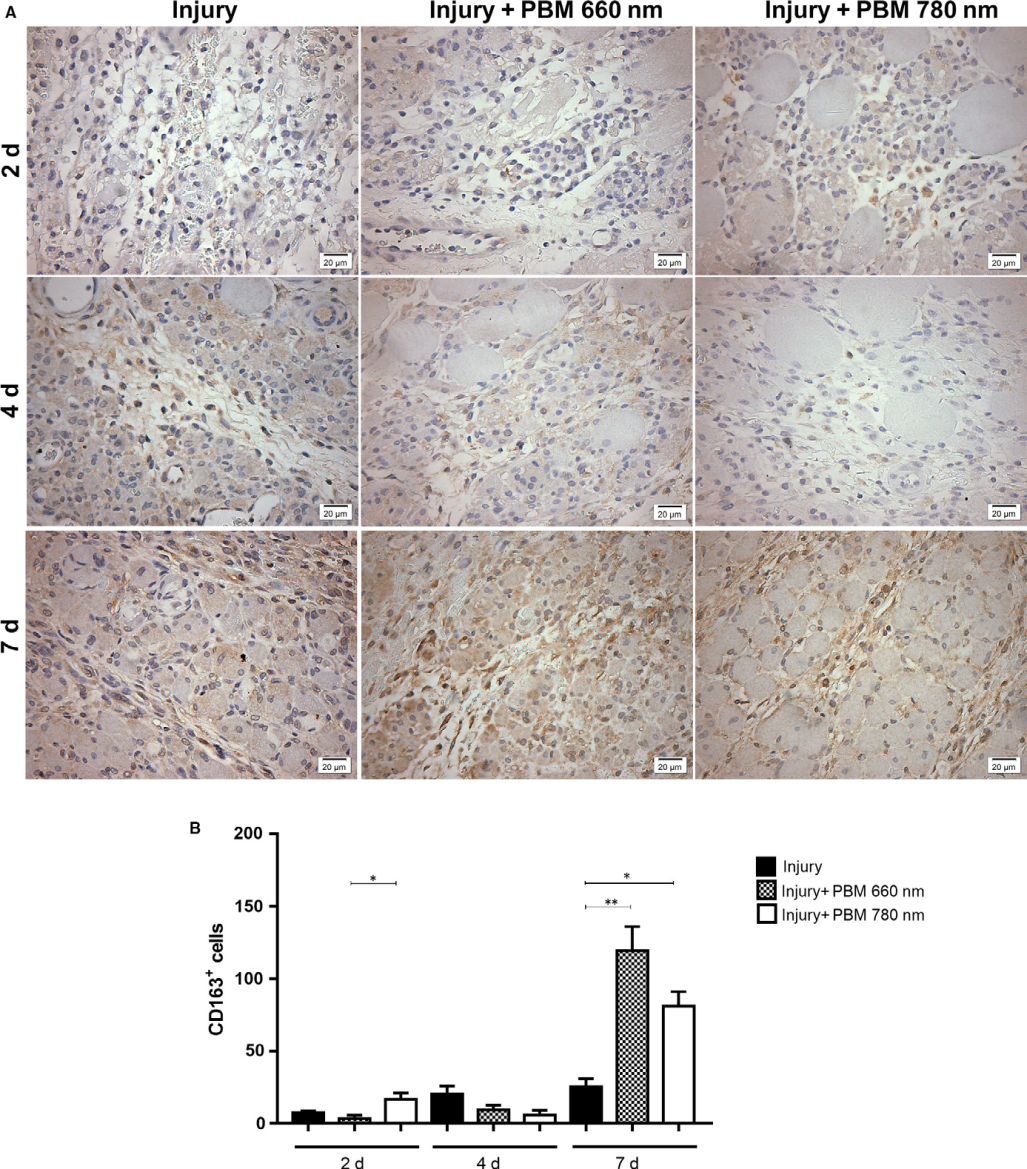
Immunohistochemical evaluation of CD163^+^ macrophages infiltration. Representative images (A) and quantitative analysis (B). The number of CD163^+^ cells was evaluated in injured muscle with or without PBM treatment in different periods (2, 4 and 7 d). A significant increase was found in the PBM 780 nm group on Day 2 in comparison to the PBM 660 nm group (*P* < .05). On Day 7, a significant increase was found in the PBM 660 and 780 nm groups in comparison to the injury group (*P* < .01 and *P* < .05, respectively. (Original magnification: 400×). Data expressed as mean ± SEM. ANOVA/Tukey's test; *P < .05; **P < .01. PBM, photobiomodulation

No CD163^+^ macrophages were found in the control group (Figure S1). Two days after injury, all groups subjected to cryoinjury exhibited a small number of CD163^+^ macrophages (Figure 5), but the number of CD163^+^ macrophages was significantly higher in the PBM 780 nm group compared to the PBM 660 nm group (*P* = .0243). On Day 4 after cryoinjury, no significant differences were found among all groups. On Day 7, a significant increase in the number of CD163^+^ macrophages was found in the PBM 660 nm and PBM 780 nm groups compared to the injury group (*P* = .0044 and *P* = .0259, respectively; Figure 5), whereas no significant differences were found between the 2 PBM groups.

### 3.3 mRNA expression levels of *TNF‐*α, *TGF‐*β and *IL‐6*

The control group was used as reference to evaluate the relative mRNA expression level of each gene. Differences in *TNF‐*α, *TGF‐*β and *IL‐6* mRNA expression were found in all periods.

#### 3.3.1 *IL‐6*

**Figure 6:**
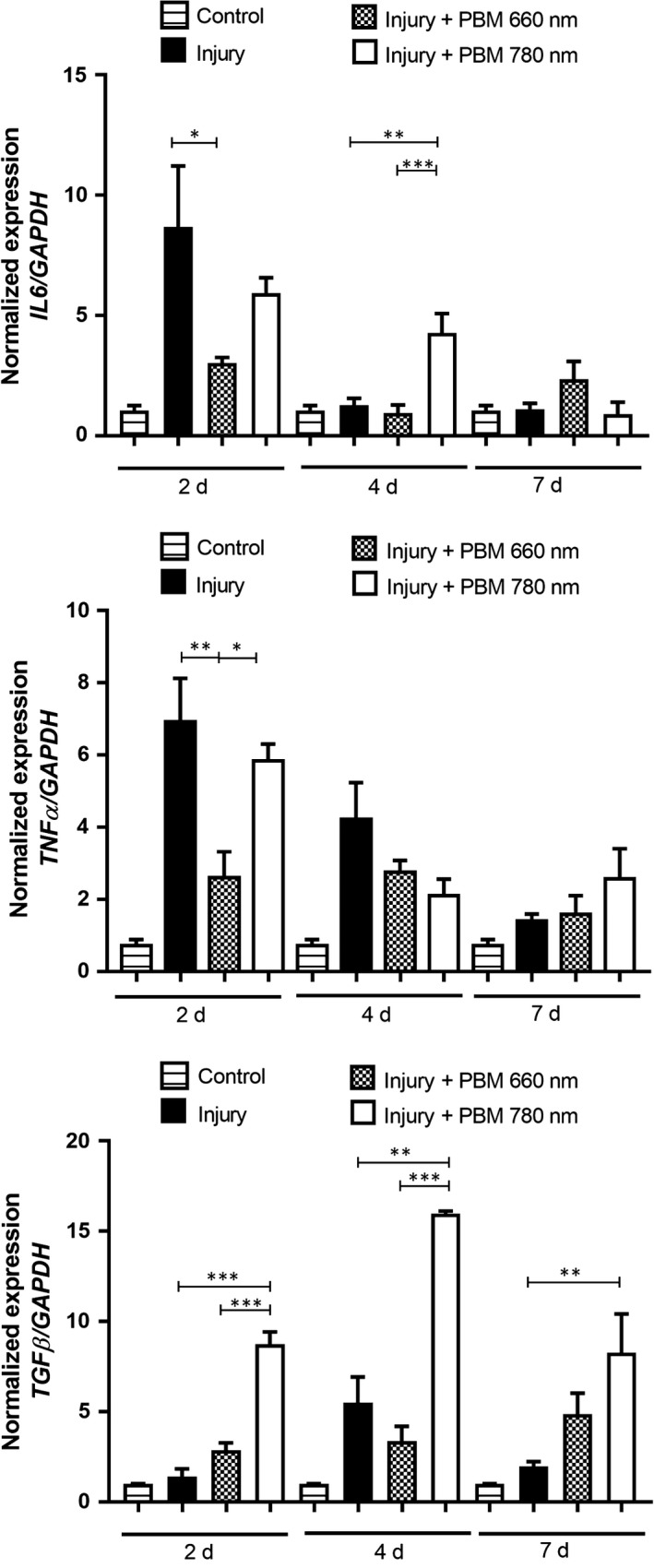
*IL‐6*,*TNF‐*α and *TGF‐*β mRNA expression in control, injury, PBM 660 nm and PBM 780 nm 2, 4 and 7 d after injury. Data expressed as mean ± SEM (ANOVA/Tukey's test; *P < .05; **P < .01; ***P < .001). PBM, photobiomodulation

*IL‐6* mRNA expression in the injured group reached its peak on Day 2 (Figure 6). Both PBM treatments were able to decrease the mRNA expression of *IL‐6*. However, the reduction was more evident in the PBM 660 nm group (*P* = .0390) compared to the PBM 780 nm group (Figure 6). On Day 4, the PBM 780 nm group exhibited a significant increase in *IL‐6* mRNA expression compared to the injury and PBM 660 nm groups (*P* = .0014 and *P* = .0006; Figure 6). No significant differences were found among the experimental groups on Day 7.

#### 3.3.2 *TNF‐*α

The highest *TNF‐*α mRNA expression level was found in the injury group on Day 2. *TNF‐*α mRNA expression was significantly lower in the PBM 660 nm group compared to both the injury and PBM 780 nm groups (*P* = .0073 and *P* = .0262, respectively), with no significant difference between the latter 2 groups (Figure 6). No differences were found among the groups on Days 4 and 7.

#### 3.3.3 *TGF‐*β

The highest *TGF‐*β mRNA expression level in the injury group was found on Day 4. On Days 2 and 4, the PBM 780 nm group exhibited significantly higher *TGF‐*β mRNA expression when compared to the injury and PBM 660 nm groups (*P* = .0003, *P* = .0005, *P* = .0022, *P* = .0008). A significant difference was observed between PBM 780 nm group and injury group (*P* = .0050) on Day 7.

## 4 DISCUSSION

To the best of our knowledge, this is the first study to show that PBM at wavelengths of both 660 and 780 nm can modify the macrophage phenotypic profile during the muscle repair process, with favourable modulation of the inflammatory and regenerative processes following an acute muscle injury. In the inflammatory phase of muscle tissue repair, the effects were more accentuated in the group treated with PBM at 780 nm, as this wavelength led to a decrease in myonecrosis and in the number of both inflammatory cells and CD68^+^ macrophages (M1 phenotype), which was accompanied by an increase in the number of CD206^+^ macrophages (M2 phenotype) and expression of *TGF‐*β mRNA 2 days after injury. In the transition phase between inflammation and repair (4 days after injury),^1^ the group treated with 780 nm continued to exhibit lower myonecrosis and inflammatory cells as well as greater expression of *IL‐6* and *TGF‐*β mRNA in comparison to the injured group. Seven days after injury, the group treated with PBM at 780 nm exhibited a larger number of blood vessels, new muscle fibres, CD163^+^ macrophages (M2) and greater expression of *TGF‐*β mRNA, which is important to terminal differentiation and growth during muscle repair.^1^

The experimental model used in the present study was able to induce a muscle repair profile similar to that previously described.^1^, ^2^ Cryoinjury, which can be considered an acute injury^1^, ^12^, ^13^, ^16^, ^17^, ^21^ provoked myonecrosis and the infiltration of inflammatory cells in the injured area on Day 2. A large number of CD68^+^ macrophages (M1) and small number of CD206^+^ and CD163^+^ (M2) macrophages were also found 2 days after injury, which is in accordance with previous descriptions (for review, see^1^). Here, the proinflammatory cytokines *IL‐6* and *TNF‐*α mRNA were also highly expressed on Day 2, as described previously.^1^ Four days after injury, reductions were found in myonecrosis, the infiltration of inflammatory, cells and CD68^+^ (M1) macrophages as well as the expression of *IL‐6* and *TNF‐*α mRNA. Interestingly, the number of CD206^+^ (M2) macrophages reached its peak, demonstrating the switch to the reparative phase, as confirmed by the higher expression of *TGF‐*β mRNA, which is an anti‐inflammatory cytokine involved in the regulation of M1/M2 polarization. In the last experimental period examined (Day 7), the amount of myonecrosis, number of inflammatory cells, CD68^+^ (M1) and CD206^+^ (M2) macrophages as well as the expression of *IL‐6, TNF‐*α and *TGF‐*β mRNA were lower compared with previous periods (Day 2 and 4). Moreover, the number of CD163^+^ (M2) macrophages and immature fibres reached its peak, once again corroborating previous studies that correlate this phenotype with the differentiation phase of the muscle repair process.^1^

It is noteworthy that, during all stages of the muscle repair process, the inflammatory infiltrate is formed by macrophages in different stages of polarization and activation. The markers associated with the M2 phenotypes and M1 phenotype can be expressed simultaneously, but it remains unclear whether an individual cell is capable of adopting different phenotypes at different times.^1^, ^2^, ^4^, ^24^, ^25^, ^26^ Therefore, the expression of the phenotype markers and functional state of macrophages are not as strict as in the case of lineage markers of other immune cells.^25^

The chronological predominance of cells associated with the inflammatory pattern or repair pattern is what likely determines the evolution of the process.^1^, ^4^, ^5^ In the first days after injury, M1 (CD68^+^) macrophages predominate probably due to stimuli received from the IFN‐γ and TNF‐α enriched environment.^1^ CD206^+^ macrophages peak on Day 4 after a muscle injury^4^ and these cells mainly express TGFβ, arginase, chemokine motif ligand 18 (CCL18), VEGF‐A, platelet‐derived growth factor (PDGF) and insulin‐like growth factor (IGF) as well as other growth factors, which favours tissue repair.^1^, ^25^, ^26^ CD206 expression can be increased by IL‐4, the granulocyte macrophage colony‐stimulating factor (GM‐CSF) and TGF‐β.^1^, ^25^, ^26^ CD163^+^ macrophages predominate during the terminal differentiation of the repair process, which occurs between the 4th and 7th day after an injury.^1^ The expression of these macrophages is highly influenced by cytokines, being downregulated by TNF‐α, TGF‐β and IFN‐γ and induced by M‐CSF, IL‐6, IL‐10 and glucocorticoids.^1^, ^25^ Besides internalizing hemoglobin and haptaglobin complexes, CD163^+^ macrophages also express IL‐10, which potentiates the anti‐inflammatory effect.^1^ In the present study, TNF‐α expression in the injury group peaked on Day 2, diminishing thereafter, and the expression of TGF‐β peaked on Day 4, diminishing thereafter. This environment enriched with cytokines favours the increase in the presence of CD68^+^ macrophages in the first 2 days, the increase in CD206^+^ macrophages observed on Day 4 (Figure 4) and the elevated expression of CD163 observed on Day 7 (Figure 5).

The results of the present study also demonstrate that PBM treatment using red light (660 nm) induced a reduction in inflammatory infiltrate, myonecrosis, the number of CD68^+^ (M1) macrophages and the mRNA expression of *TNF‐*α after 2 days and promoted an increase in the number of immature muscle fibres and CD163^+^ (M2) macrophages after 7 days. Using PBM at 660 nm (20 mW and 1.6 J) with the same experimental model, De Souza et al^12^ and Mesquita‐Ferrari et al^16^ also found a reduction in myonecrosis at 7 days as well as a reduction in *TNF‐*α mRNA expression after 1 and 7 days. *TNF‐*α is a proinflammatory cytokine that stimulates myogenic cell proliferation, but also inhibits its differentiation and fusion.^3^, ^24^ Therefore, the decrease in *TNF‐*α expression during the experimental periods could explain the decrease in myonecrosis on Day 2 and the increase in immature muscle fibres on Day 7 in the group treated with PBM at 660 nm. *TNF‐*α also downregulates CD163 expression,^1^ which may have contributed to the increase in CD163^+^ (M2) macrophages at 7 days in this group.

The results obtained with 780 nm concerning morphological aspects and the mRNA expression of cytokines reported in the first paragraph of this section are also in agreement with previous findings. Alves et al^13^ administered PBM at 780 nm (40 mW and 3.2 J) using the same experimental model and found a decrease in inflammatory infiltrate and myonecrosis after 1 day, an increase in the number of blood vessels after 3 and 7 days as well as an increase in the number of immature muscle fibres after 7 days. Brunelli et al^14^ also found that PBM at 780 nm (20 and 40 mW; 0.4 and 2.0 J, respectively) caused a decrease in inflammatory infiltrate in rat muscle 7 days following injury. On the other hand, using NIR 808 nm PBM (30 mW and 1.4 J), Assis et al^19^ found decreased expression of *TGF‐*β mRNA 4 days following cryoinjury. In the present study, PBM at 780 nm (70 mW and 8 J) led to an increase in *TGF‐*β mRNA after 2 and 4 days. This difference could be related to the different amounts of energy delivered in the 2 studies.^10^, ^11^ *TGF‐*β is an anti‐inflammatory cytokine capable of regulating the M1‐M2 polarization state and is also an important growth factor involved in the modulation of the proliferation and differentiation of muscle cells, since *TGF‐*β inhibits the transcriptional activity of myogenin, which is an important myogenic regulatory factor.^1^, ^3^, ^25^ *TGF‐*β also modulates fibroblast collagen production and the up‐regulation of this growth factor can lead to the transient formation of fibrosis or scar tissue.^26^ In the present study, *TGF‐*β mRNA reached its peak at 4 days in the injured group and in the group treated with PBM at 780 nm. On Day 7, a significant difference in *TGF‐*β mRNA was found only in PBM 780 nm group, indicating also a temporal increase in the gene expression of this cytokine. The increased expression of *TGF‐*β during the 7 days in the 780 nm group could explain the decrease in myonecrosis and the increase in blood vessels in this group.

Regarding *IL‐6*, Alves et al^18^ also found an increase in *IL‐6* mRNA expression 14 days after injury in muscles treated with PBM at 780 nm (40 mW and 3.2 J). *IL‐6* is manly produced by macrophages and other components of skeletal muscle (endothelial cells, fibroblasts and muscle cells) and plays an important role in macrophage infiltration as well as the proliferation and differentiation of myogenic precursor cells.^3^, ^27^, ^28^ Here, *IL‐6* up‐regulation could be responsible for the increased number of immature muscle fibres found in the group treated with PBM at 780 nm.

The multiple effects reported above are related to the fact that PBM outcomes depend on the absorption of light by chromophores.^9^, ^10^, ^11^ Cytochrome c‐oxidase (unit IV in the mitochondrial respiratory chain present in all eukaryotic cells) is one of the main chromophores of light photons from the red and NIR spectral regions.^9^, ^10^, ^11^ The absorption of light by cytochrome c‐oxidase promotes the dissociation of its inhibitory nitric oxide, which leads to an increase in electron transport, mitochondrial membrane potential, ATP generation and the activation of other signalling pathways.^9^, ^10^, ^11^ The results of these secondary effects include the activation of many transcription factors, which could explain the effects of PBM on cell proliferation and survival, protein synthesis and the activation of anti‐inflammatory and antioxidant pathways, although the mechanism of action of PBM is yet to be fully described.^9^, ^10^, ^11^

An important concept regards the therapeutic window for PBM dosimetry. Dosimetric parameters, such as wavelength, power density, energy density, frequency of irradiation, operation regime and interval between consecutive irradiations, are fundamental to achieving the desired results. In the present study, red and NIR light with the same dosimetric parameters were compared and the results were better for NIR light (780 nm laser). The explanation for this difference could reside in the amount of light that actually reaches the cells, which is subject to light wavelength as well as the scattering and reflection properties of tissues.^9^, ^10^, ^11^, ^29^

The effects of PBM on macrophage phenotypes are beginning to be described in cell cultures studies,^10^, ^30^, ^31^, ^32^, ^33^, ^34^ evidencing that this therapeutic modality, especially using the NIR wavelength, is capable of altering the polarization of these cells in vitro. In vivo experiments have demonstrated that PBM (808 nm) can shift the phenotype of brain microglial polarization from the pro‐inflammatory phenotype (M1) to the anti‐inflammatory (M2) phenotype after ischemic stroke, promoting cortical neurogenesis.^35^ There are also reports of the ability of red and NIR PBM to modulate the macrophage/microglia phenotype, leading to a predominance of M2 macrophages associated with better recovery of the spinal cord and peripheral nerves after a spinal cord injury^36^, ^37^ and spared nerve injury,^38^ respectively.

In the present study, treatment with PBM at both wavelengths was able to decrease the amount of M1 macrophages 2 days after injury and increase the number of CD163^+^ (M2) macrophages 7 days after injury. However, only treatment with NIR PBM was able to increase the number of CD206^+^ M2 macrophages after 2 days. These results demonstrate that the treatment with PBM can modulate the inflammatory phase, optimize the transition from the inflammatory to regenerative phase (mainly with NIR light) and improve the final step of regeneration, thereby enhancing the tissue repair processes. Moreover, all these events could be correlated with the presence of distinct macrophage phenotypes.

Although the data described above strongly suggest that PBM can modulate macrophage phenotypes and muscle regeneration after an acute injury, further analyses involving other injury methods and, especially, humans are essential to gaining a better understanding of the role of this therapeutic tool in muscle regeneration.

## 5 CONCLUSION

As macrophage phenotype and function have been suggested to be critical and determinant in downstream outcomes in regenerative medicine^6^, ^7^ and muscle therapies,^1^, ^3^, ^4^ the evidence presented herein that PBM is a useful tool for accelerating the skeletal muscle repair process by modulating macrophage phenotypes can improve our understanding of the mechanism of action of this therapeutic modality and also indicates new possibilities for macrophage‐based therapies.

## CONFLICT OF INTEREST

The authors have no conflict of interests to disclose.

## AUTHOR CONTRIBUTION

KPSF, MFSDR and NHCS conceived and design the experiments. NHCS, MFSDR, BGR, EMSJ conducted the experiments. SKB, FDN and CMF contributed reagents/materials/analysis tools. NHCS, RAMF, MFSDR, DFTS, ANA, MPG and KPSF analyzed the results and wrote the manuscript. All authors reviewed the manuscript.
