# Supplementary figures and images for: Photobiomodulation and different macrophages phenotypes during muscle tissue repair
Source: J Cell Mol Med. 2018 Jul 19;22(10):4922–34. doi: 10.1111/jcmm.13757 (PMC6156453; doi:10.1111/jcmm.13757)

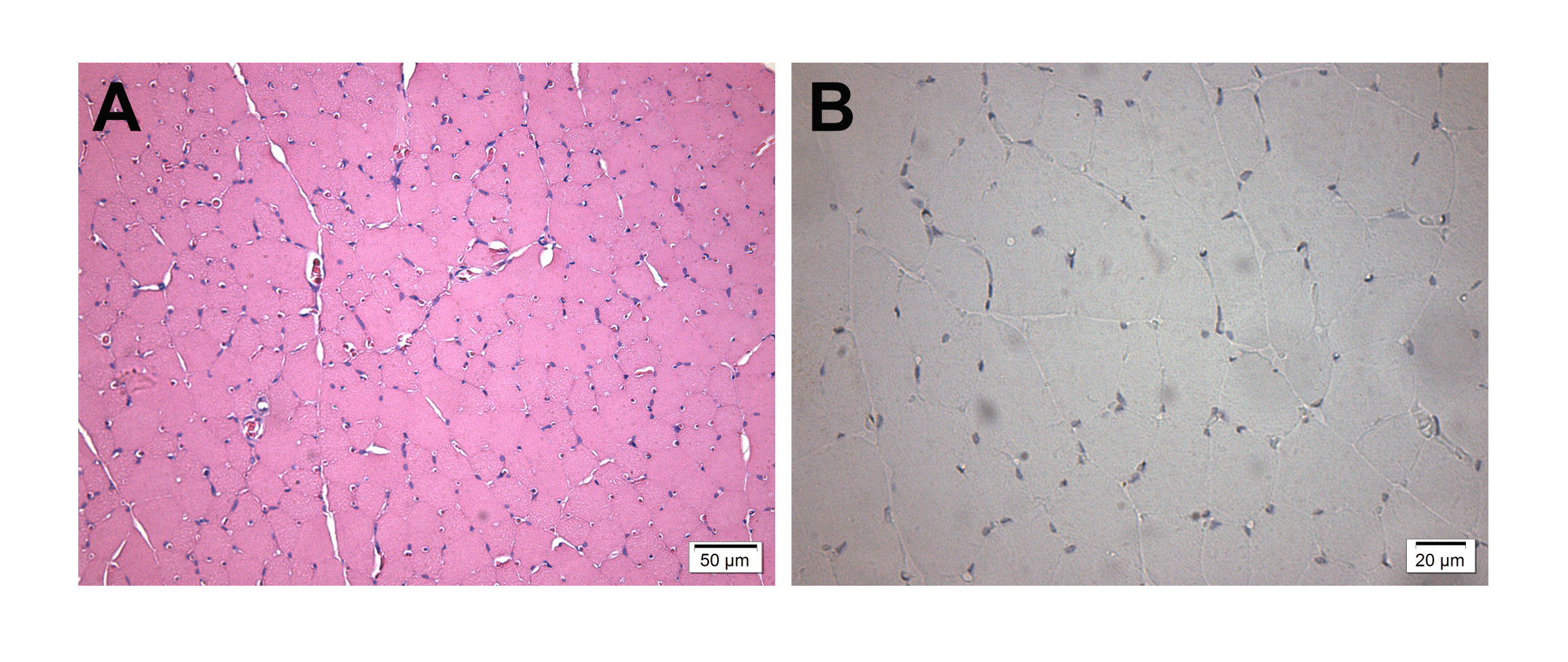

Supplement: Supplementary file 1 [file JCMM-22-4922-s001.tif]

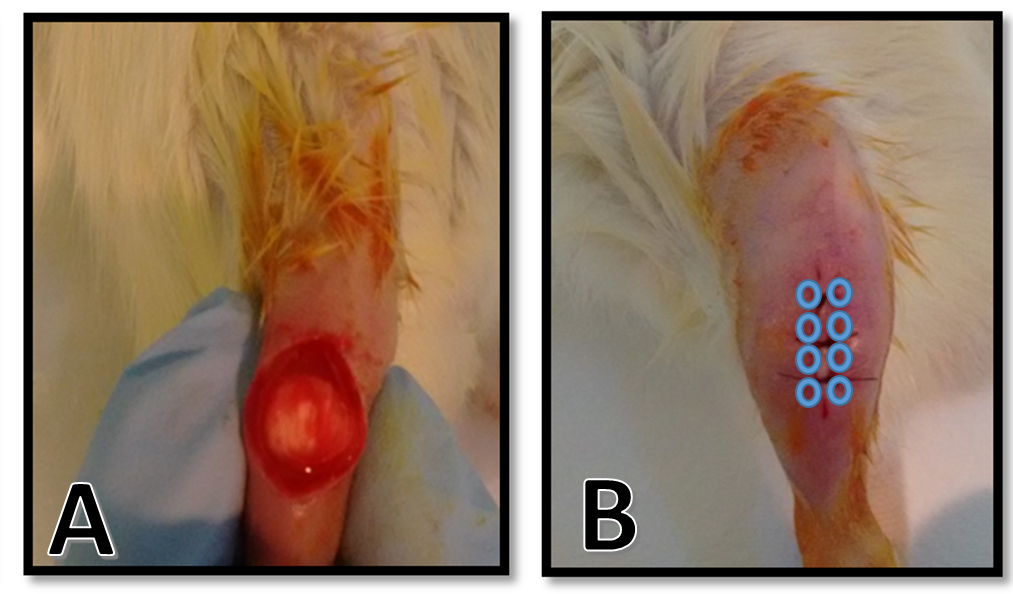

Supplement: Supplementary file 2 [file JCMM-22-4922-s002.tif]
