# Supplementary material for: Photobiomodulation and different macrophages phenotypes during muscle tissue repair
Source: J Cell Mol Med. 2018 Jul 19;22(10):4922–34. doi: 10.1111/jcmm.13757 (PMC6156453; doi:10.1111/jcmm.13757)
Supplement: Supplementary file 3 [file JCMM-22-4922-s003.docx]

**Supplemental Figure 1.** Representative images of the morphological **(A)** and immnunohistochemical evaluation of CD68, CD206 and CD163 expression **(B)** in the control group.

**Supplemental Figure 2.** Cryoinjury procedure **(A)** and PBM treatment protocol – 8 blue circles represent areas that received PBM treatment **(B)**.
